# New Method of Preparing the Spinal Cord for Microscopic Sections

**Published:** 1881-10

**Authors:** 


### NEW METHOD OF PREPARING THE SPINAL CORD FOR MICROSCOPIC SECTIONS.

The following method of hardening the spinal cord for microscopic sections has been highly recommended by Dr. M. Debove:

Place the cord in a 4 per cent. solution of bichromate of ammonia for three weeks, then in a solution of phenic gum for three days, and for three days more in alcohol. Sections may then be cut with great facility. They should be placed in water to prevent curling. They are then immersed in a saturated solution of picric acid for twenty-four hours, and colored with carmine for about twenty minutes, the picric acid acting as a mordant. —*Archives de Neurologie, July*, 1880.

